# Characterization and in-depth genome analysis of a halotolerant probiotic bacterium *Paenibacillus* sp. S-12, a multifarious bacterium isolated from *Rauvolfia serpentina*

**DOI:** 10.1186/s12866-023-02939-1

**Published:** 2023-07-18

**Authors:** Rajnish Prakash Singh, Kiran Kumari, Parva Kumar Sharma, Ying Ma

**Affiliations:** 1 Department of Biotechnology, Jaypee Institute of Information Technology, Noida, India; 2 Department of Bioengineering and Biotechnology, Birla Institute of Technology, Mesra, Ranchi India; 3 Department of Plant Sciences and Landscape Architecture, University of Maryland, College Park, MD-20742 USA; 4 College of Resources and Environment, Southwest University, Chongqing, China

**Keywords:** *Paenibacillus*, Genome, Probiotics, Pangenome, CAZymes

## Abstract

**Background:**

Members of *Paenibacillus* genus from diverse habitats have attracted great attention due to their multifarious properties. Considering that members of this genus are mostly free-living in soil, we characterized the genome of a halotolerant environmental isolate belonging to the genus *Paenibacillus*. The genome mining unravelled the presence of CAZymes, probiotic, and stress-protected genes that suggested strain S-12 for industrial and agricultural purposes.

**Results:**

Molecular identification by 16 S rRNA gene sequencing showed its closest match to other *Paenibacillus* species. The complete genome size of S-12 was 5.69 Mb, with a GC-content 46.5%. The genome analysis of S-12 unravelled the presence of an open reading frame (ORF) encoding the functions related to environmental stress tolerance, adhesion processes, multidrug efflux systems, and heavy metal resistance. Genome annotation identified the various genes for chemotaxis, flagellar motility, and biofilm production, illustrating its strong colonization ability.

**Conclusion:**

The current findings provides the in-depth investigation of a probiotic *Paenibacillus* bacterium that possessed various genome features that enable the bacterium to survive under diverse conditions. The strain shows the strong ability for probiotic application purposes.

**Supplementary Information:**

The online version contains supplementary material available at 10.1186/s12866-023-02939-1.

## Background

*Paenibacillus* genus was observed in 1993 with an estimated 11 species belonging to the genus *Bacillus* [1]. Many new members of this genus have been discovered and so far, more than 150 species have been identified. The identified members are endospore-forming, facultative anaerobic, rod-shaped, and motile [2, 3]. The spore forming ability helps *Paenibacillus* species to persist for a long period, which makes it a unique bacterium as compared to other bacterial strains in the environment [4, 5]. The reported members are morphologically, physiologically, and biochemically diverse and are present in environments like water [6], soil [7], insect larvae [8], and human feces [3]. Initially, members of this genus were reported to enhance plant growth such as *Paenibacillus polymyxa* [9]. Recently *Paenibacillus* strains showed their ability for silver nanoparticle production [10], ginsenoside transformation [11], and ginsenoside Rd production [12].

Besides their ability to plant protection, lipopetide producing *Paennibacillus* spp. has achieved great attention for the treatment of drug-resistant and human bacterial pathogens [13]. Similarly, tridecaptins produced by *Paenibacillus* spp. showed strong inhibitory activity against multidrug-resistant bacterial strains by binding to lipid II on the bacterial inner membrane leading to disruption of proton motive force [14]. The produced tridecaptins showed low cytotoxicity and higher stability in human plasma. The other member *P. larva*was identified as spreading epizootic disease by American Foulbrood (AFB) [8]. These bacteria were equipped with various carbohydrate (cellulose, starch, and xylan) hydrolyzing enzymes [15, 16]. Genome analysis identified the genes for antimicrobial and volatile organic compounds, siderophore production, phosphate transport, and indole acetic acid (IAA) synthesis etc. in *P.yonginensis* DCY84T [17].

Among the reported *Paenibacillus* strains, *P. polymyxa* showed probiotic features. The livestock feed isolated bacterium *P. polymyxa* JB-0501 was exploited as a potent probiotic candidate following *in-vitro* evaluation [18]. Another bacterium *P. polymyxa* strain HGA4C isolated from fish gut demonstrated the antibacterial and probiotic features under *in-vitro* and *in-vivo* conditions [19]. The bacterium *Paenibacillus* sp. Aloe-11 secretes the extracellular enzymes for metabolization of complex polysaccharides and colonized the chicken intestine [20]. A strain of *P. polymyxa* is marketed as a potent probiotic for aquaculture purposes under the Biostart® trade name [21].

Besides genome sequencing, comparative genomics provides valuable information on the gene repertoires associated with metabolic activities and their adaptability to the environment [22, 23]. Additionally, comparative genomics tells us about other processes like gene duplication, increased flux expansion through horizontal gene transfer (HGT), gene loss, and genome reduction [24]. Members of the *Paenibacillus* genus showed potent environmental adaptability and a broad genome size [25, 26]. However, the detailed information about the adaptability of this genus based on the genome dynamics is still unclear. Till now, more than 100 *Paenibacillus* genomes have been sequenced, which require in-depth analysis to understand the gene repertories linked with adaptability to diverse environments.

Several features like bacterial colonization [27], antagonistic activity [28] and the ability to confer induced resistance [29] are the main reported mechanism of biological control. Previous studies [30, 31] showed that *P. polymyxa* secretes various metabolites including polymyxins, fusaricidins, and other antibiotics belonging to the category of antibacterial and antifungal activities. The different types and amounts of antibiotics affect the antimicrobial spectra and their biocontrol efficacy. The biocontrol ability might be due to the release of various volatile organic compounds mainly 2,3-butanediol, isoprene, butyl acetate, and n-hexadecane etc. into the surrounding environment which imparts a protective effect on plants [29, 32]. Some of the *Paenibacillus* spp. is well known for their ability to induce plant growth [8, 9]. Recently, Furlan et al. reported a polyethylene degrading bacterium *P. aquistagni* strain DK1 and identified an alkB-like gene and other structural motifs related to alkane hydrolases such as His boxes, and HYG motif [33].

In the last few decades, biosynthetic gene clusters (BGCs) related to secondary metabolite production has been characterized in microbes [34]. Identification of these BGCs explores the information about the genes encoding key signature enzymes and antimicrobials proteins (AMPs) [34]. These AMPs include polymyxin, paenibacterin, and lipopeptides which showed strong inhibitory activity against bacteria, fungi and even cancer cells [35, 36]. These BGCs includes the NRPSs (nonribosomal peptide synthetases), PKSs (polyketide synthase) and RiPPs (ribosomally synthesized and post-translationally modified peptides) [37]. The exploration of BGCs in microbes provides important information about the distribution of various genes for secondary metabolites production and the identification of potent industrially relevant strains with novel and/or improved functionality [38]. This information can be used for developing industrial-relevant strains for their wide applications. The secondary metabolites are required for the growth and development of the host as well as confer protection against infections [39]. Considering the great demand for the identification of new bacterial strains with the ability to control the growth of pathogenic microbes, we explored the genome annotation of *Paenibacillus* sp. S-12 to identify BGCs and AMPs. The use of AMPs in the food industry as natural antimicrobial agents is generally recognized as safe (GRAS) and promises safety and food product quality [40, 41].

Carbohydrate-active enzymes (CAZymes) constitute broader enzyme group involved in the degradation as well as rearrangement of glycosidic bonds in carbohydrates [42]. These CAZymes are classified majorly into glycosyl transferases (GTs), glycoside hydrolases (GHs), polysaccharide lyases (PLs), carbohydrate esterases (CEs), carbohydrate-binding modules (CBMs), and auxillary activities (AAs) [43]. Previously, CAZymes were noted by the genome annotation of *Paenibacillus* sp. JDR-2, *P. mucilaginous* KNP414, and *P. terrae* HPL-003. Recently, carbohydrate-active enzymes (CAZymes) as well as enzymes responsible for woody biomass degradation in termite gut were identified in the genome of *P. polymyxa* A18 [44]. However, the increasingly industrial applications of CAZymes demand the exploration of new microbial resources for more diverse CAZymes.

The gene repertoires of the genus *Paenibacillus* are in incessant flux and the genome size of *Paenibacillus* shows high plasticity, therefore, we explored the genomic analysis of newly isolated bacterium *Paenibacillus* sp. S-12. The aim of the work was to carry out the physiological, biochemical and genomic characterization of halotolerant *Paenibacillus* sp. S-12. The strain possesses several beneficial gene features such as presence of CAZymes, which make it an important microorganism for use in the area of environmental biotechnology and other industrial applications.

## Methodology

### Bacteria isolation

The bacterial strain S-12 was isolated from the rhizospheric soil of *Rauvolfia serpentine* growing around the salt belt region of Ranchi, India (23.41° N, 85.43° E). The attached soil was brought to the laboratory in ziplog bags and serially diluted in 1X PBS (phosphate buffer saline) solution. The serially diluted samples were plated on the sterile LB-agar plate amended with different salt concentrations (2 to 8% NaCl) and incubated at 37 °C overnight. One colony showing luxuriant growth on 6% salt-amended plate was further used for detailed characterization. Glycerol stock (20% v/v) was used for the preservation of bacterial culture.

### Biochemical characterization

Gram staining of strain S-12 was done by bacterial Gram-stain kit (Himedia, India). The strain S-12 was tested for biochemical characteristics such as IMViC (Indole, Methyl Red, and Voges Proskauer), citrate utilization, catalase and oxidase activity following the standard protocol. The strain was tested for pH tolerance (pH 5 to 10) and temperature tolerance (25 ℃ to 45 ℃). The capacity of utilizing different carbon sources was tested by KB009-HiCarbo Kit (Himedia, India). For the carbon utilization test, strain S-12 was grown in tryptic soya broth (Himedia, India) up to 0.6 at 600 nm. Following growth, 50 µl of bacterial suspension was aseptically transferred into each well and kept for incubation for 24 to 48 h at 37 ℃. The interpretation of results was recorded following observation of colour change and as per the instruction sheet supplied with the kit. The antagonistic activity against bacterial strains (*E. coli, P. aeruginosa, B. subtilis, S. aureus*), and fungal strains (*Aspergillus niger, Microsporum gypseum, H. gypsium* and *Penicillium citrium*) was performed by well diffusion method [45].

### Antibiotics sensitivity test

The strain S-12 was tested for their susceptibility against diverse antibiotics namely erythromycin (15 µg), ampicillin (10 µg), kanamycin (30 µg), tetracycline (30 µg), ciprofloxacin (5 µg), gentamicin (10 µg), fluconazole (25 µg), streptomycin (10 µg), vancomycin (30 µg), and voriconazole (15 µg) following CLSI (Clinical and Laboratory Standards Institute) instruction. The plates were incubated for 24–48 h at 37 °C and the result was interpreted by measuring the zone of inhibition (ZOI).

### Scanning electron microscopy

Scanning electron microscopy (SEM) was performed to observe the morphology of the test isolate. The overnight grown culture was pellet down in 2 ml Eppendorf tubes and washed three times with PBS (phosphate buffer solution). The pellet was fixed in the mixture of glutaraldehyde and buffer in the ratio of 1:9 at pH 7.2. After mixing, the sample was kept on ice for 45–60 min. The suspension was centrifuged and the pellet was dehydrated in ascending grades of ethyl alcohol. The dried sample was transferred in a carbon stub and further moved for SEM analysis (JSM-6390LV, Jeol, Japan) at 500-1000X magnification.

### Molecular identification

The genomic DNA of S-12 was extracted from the mid-exponential phase using Qiagen Kit (Qiagen, Germany). The S-12 strain was identified as *Paenibacillus* sp. by amplification and sequencing of the 16 S rRNA gene using the universal 27 F1 and 1492 R2 primers set [46] .The condition for PCR includes an initial denaturation at 95 °C for 5 min, followed by 30 cycles at 95 °C for 30 s, 55 °C for 40 s, and 72 °C for 40 s, with the final step at 72 °C for 10 min. The PCR amplification was performed in a Gene-Amp PCR system 2400 (Applied Biosystem, Thermo Scientific, USA). The amplified product was subjected to 1% gel electrophoresis, stained with ethidium bromide and the product was sequenced at Eurofins Genomic Labs Ltd. (Eurofins, India). The obtained sequence was analyzed for BLAST homology at the public available NCBI database http://www.ncbi.nlm.nih.gov/BLAST. The Clustal Omega (https://www.ebi.ac.uk/Tools/msa/clustalo) was sued for sequence alignment and the phylogenetic tree was constructed by MEGA 7.0 [47]. The 16 S rRNA sequence was submitted to the Genbank database for obtaining an accession number.

### Screening for probiotic propensities

The test isolate S-12 was grown in the TSB (tryptic soya broth) (Himedia, India) and late log phase cells were collected by centrifugation at 8,000 rpm for 15 min at 4 ℃. The obtained cells were washed with sterile 1X PBS buffer (pH 7.4) and adjusted to a cell density of 1 × 10^7^CFU/ml. The acid tolerance was tested by inculcating in the sterile TSB medium with different pH (2.0 & 7.0) and kept for incubation at 37 ℃ for 90 min. The cells count was performed by plating the 100 µl of serially diluted cell suspension on sterile TSA (tryptic soya agar) plates. Similarly, bacterial cells were inoculated into the TSB medium supplemented with 0.2%, 0.3% and 0.4% bile (Himedia, India) to test the bile tolerance [48]. Following, incubation at 37 ℃ for 24 h, the viability was assessed as mentioned above. The medium without bile was used as a control.

### Biofilm formation test

The biofilm formation ability of strain S-12 was tested by the standard crystal violet (CV) staining method [49]. The bacterial population of S-12 was adjusted to 10^8^CFU/ml in the TSB medium and cell suspension of 20 µl was transferred to a 96-well microtiter plate filled with 180 µl of sterile LB (Himedia, India). The control wells were filled with LB-medium only. The plates were incubated at 37 °C for 24 h under static conditions. To check the biofilm formation ability, separate plates with the above-mentioned treatment were incubated at 25, 30, 35, and 40 °C. Following incubation, medium was discarded and wells were washed with 3x PBS and filled with 200 µl of 0.1% CV (Sigma-Aldrich, USA), and incubated at room temperature for 30 min. The CV was decanted and fixing of bacteria was done by hot air stream at 60 ℃ for 1 h. The resolubilization of dye-bound cells was performed by three rinses with 200 µl of ethanol (Merck, India) per well and OD was measured at 595 nm using a microtiter plate reader. Each treatment was done in triplicate to calculate the average value and for accuracy the experiment was repeated three times.

### Endospore formation test

Endospore formation ability of S-12 was evaluated microscopically and by standard plating procedure. The test strain was grown in Difco sporulation medium (DSM: Difco nutrient broth 8 g/l, MgCl_2_ 0.49 mM, KCl 13.4 mM, Ca(NO_3_)_2_ 1 mM, MnCl_2_ 10 µM, and FeSO_4_ 1µM) for 48 h at 37 °C with constant stirring at 180 rpm. Bacteria was pelleted at 10,000 rpm for 10 min at 4 °C and washed in cold-sterile water followed by heat shock at 70 °C for 30 min to kill the vegetative cells. The sporulating cells were observed under phase-contrast microscopy.

### Genome sequencing, assembly and annotation

Genomic DNA was isolated from the QIAamp DNA Mini Kit (Qiagen, Germany). The extracted DNA sample was sequenced using an illumine MiSeq platform and the paired-end library was prepared by using the NEB Next Ultra DNA Library Prep Kit. Fast QC program was used for Quality control of Illumina reads (http://www.bioinformatics.babraham.ac.uk/projects/fastqc). The Illumina reads were used for hybrid assembly using the SPAdes version 3.15.2 [50].The transfer RNA (tRNA) and ribosomal RNA (rRNA) of the S-12 were identified using the tRNAscan-SE and RNAmmer (v1.2, http://www.cbs.dtu.dk/services/RNAmmer/) software, respectively. The genome was annotated using Prokka [51] and RAST [52] tool against the NCBI non-redundant (NR) database. The COG (clusters of orthologous groups) analysis of protein was performed by BLASTP [53]. The genes involved in biological pathways were annotated using KEGG and Blast2Go tools [54]. The analysis of orthologous gene clusters was analyzed using the Orthovenn2 program [55] using protein sequences of S-12, *P. alvei* DSM29, *P. curdlanolyticus* YK9, *P. polymyxa* ATCC842, P. *polymyxa* E681, and *P. polymyxa* SC2. The multiple alignments of the genome sequences were performed using MUSCLE [56]. The tree was constructed by NJ-method following the kimura-2 model using the MEGA 7.0 [57, 58].

### Antimicrobial and virulence analysis

The CARD database was used using a homology based approach (BLASTX) against the genome sequence of S-12 to unravel the presence of AMR genes. For searching, BLAST output was filtered with a minimum 70% identity and subject protein coverage. Similarly, the VFDB database was used against assembled genome with criteria of a minimum of 70% identity using a homology-based approach (BLASTX) to identify the virulence genes.

### Detection of secondary metabolite biosynthetic gene cluster

The number and types of secondary metabolite BGCs in the genome sequence of S-12 was identified by antiSMASH version 5.1.2 in combination with Hidden Markov Model (HMM) to detect the BGCs-like region [37, 59]. Various unknown and characterized BGCs were identified and genetic similarities in gene clusters were predicted using antiSMASH 5.1.2.

### Prediction of carbohydrate‑active enzyme (CAZymes)

To reveal the presence of various CAZymes including glycosyltransferases (GTs), glycoside hydrolases (GHs), polysaccharide lyases (PLs), carbohydrateesterases (CEs), auxiliary activities (AAs), and carbohydrate-binding modules (CBMs), the protein sequences of S-12 was annotated using the dbCAN2 server [60] and BLAST-driven DIAMOND against the CAZy database [43]. The diversity of CAZymes in closest relatives of *Paenibacillus* species was also performed to evaluate the comparative distribution.

### Pan genome-core genome analysis

Strain S-12 and its closest non-type strains were used for prediction of core and accessory genes using Roary v 3.11.2 with default setting [61] based on GFF3 file of all selected genome generated through PROKKA v 1.14.5 [51]. The strains were selected based on the higher similarity of S-12 to other closely related strains in the RAST (Rapid Annotation using Subsystem Technology). To determine the presence and absence of each core gene in selected strains, matrix was visualized with Phandango [62].

## Results

### Biochemical characterization

The isolated strain was found to be a Gram-positive, rod shaped bacterium. Among biochemical tests, it showed the negative result for indole, methyl-red and positive for voges-proskauer, citrate, oxidase and catalase (Table S1). The strain was able to grow in a wide range of pH (5 to 9) and temperature (30 to 40 ℃) (Table S1). Among the tested various carbon sources, the strain was able to utilize lactose, xylose, maltose, galactose, melibiose, sorbitol, glycerol, D-arabinose, citrate, malonate, ONPG, inulin, inositol, trehalose and mannoside etc. (Supplementary Table 1). The isolate showed a higher sensitivity (20 to 25 mm) against streptomycin, gentamicin, erythromycin, ciprofloxacin, vancomycin, and moderate sensitivity (10 to 18 mm) to ampicillin, fluconazole, tetracycline, and resistant to kanamycin, voriconazole (Supplementary Table 2). SEM analysis confirms the rod-shaped colony of the isolated bacterium (Supplementary Figure S1). Phenotypically on a motility-specific medium, the strain showed the swimming, swarming, and twitching motility behavior (Supplementary Figure S2). The test isolate showed good antagonistic activity against *E. coli*, *P. aeruginosa*, and moderate against *B. subtilis* and *S. aureus.* Against the fungal strains, strain showed good antagonistic activity against *A. niger, M. gypseum*, and moderate against *H. Gypsium* and *P. citrium* (Supplementary Table 3).

### Identification and phylogenetic analysis

**Fig. 1:**
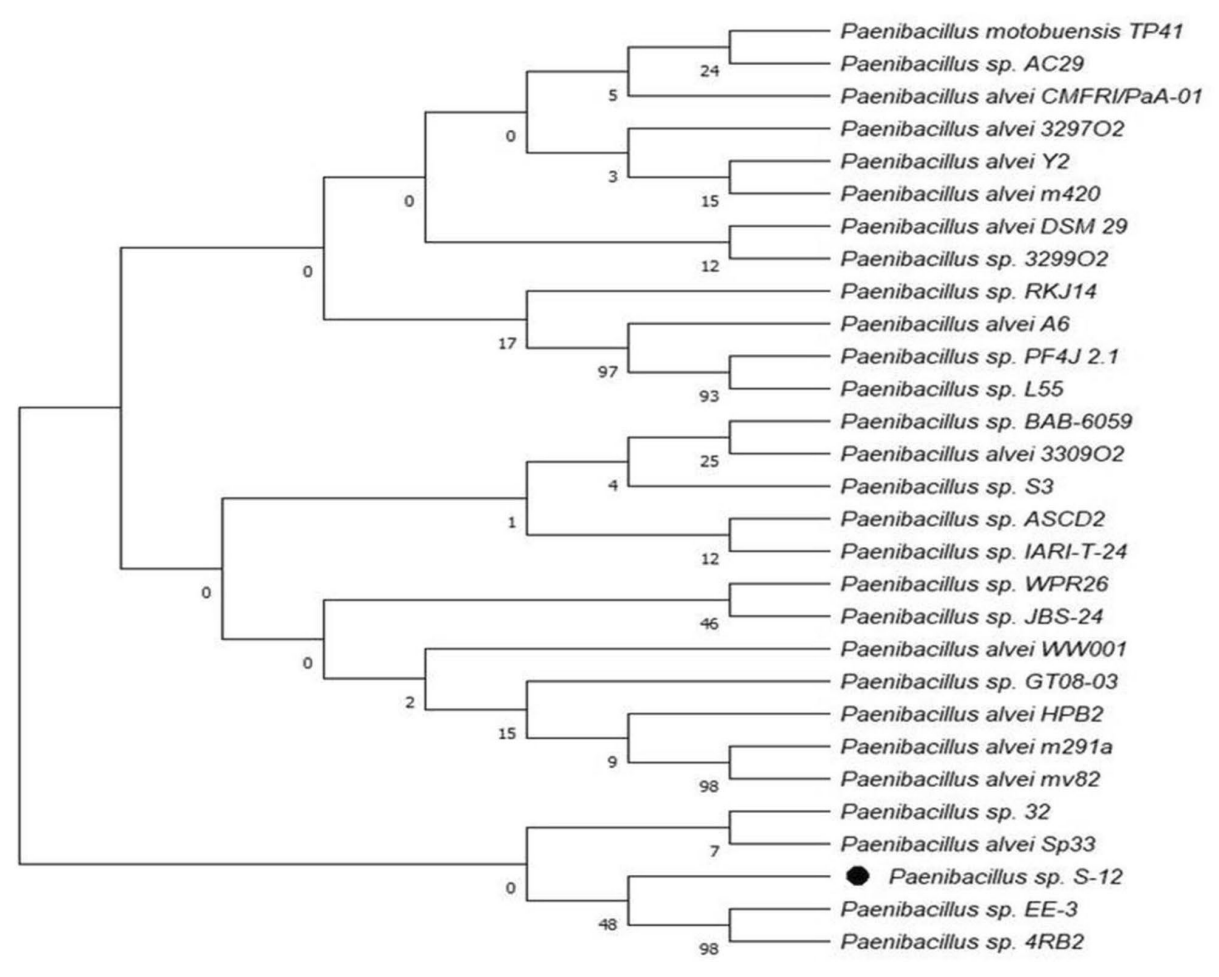
Phylogenetic tree showing relationship of *Paenibacillus* sp. S-12 to closely related bacterial strains. The 16 S rRNA gene sequence of closely related species was obtained from NCBI GenBank database. The rooted tree was obtained using Neighbor-joining method of software packages Mega version 7.0, at bootstrap value of (n = 500)

For identification at molecular level, 1.5 Kb PCR amplicon of 16 S rRNA gene was sequenced at Eurofins Genomics Pvt Ltd. (Karnataka, India), and obtained sequence was submitted to the NCBI Genbank (accession no. OM943075). Following BLAST result, the strain was identified as *Paenibacillus* sp. with the closest match of 98% similarity to *Paenibacillus* sp. 4RB2 (Fig. 1). The strain also showed its closest similarity to other *Paenibacillus* strains used in this study.

### Probiotic properties

The selection of probiotic strain involves its ability to sustain the low pH of the stomach as well as the presence of bile in the intestine. The test isolate exhibited good tolerance to low pH and bile salts. In response to low pH, there was a decrease in bacterial counts from 1.0 × 10^7^ to 4.5 × 10^4^ (Supplementary Figure S3). Following exposure to bile salts, there was no significant decrease in the viability of S-12 (Supplementary Figure S3). The genome analysis revealed the presence of genes responsible for pH homeostasis and metabolic rearrangements, general and secondary stress, which ensures its survival during gastrointestinal transit. In the S-12 genome, we identified the genes for Na^+^/H^+^ antiporter, F0/F1-ATP synthase, alcohol and lactate dehydrogenase, and amino acid decarboxylase which assist the bacteria in stress survival. Additionally, arcD genes corresponding to arginine/ornithine antiporters, glutamate decarboxylase, and arginine deaminase were also identified (Table 1). The various other proteins involved in the repair of macromolecules such as DnaK/DnaJ chaperone, GroEL, and GroES were identified in the S-12 genome.

### Biofilm formation ability

The test strain S-12 was identified as biofilm producers. The isolate displayed a moderate biofilm formation ability (OD_595_ < 0.462) at 25 °C, whereas at 30, 35, and 40 °C, it showed good biofilm (OD_595_ > 0.462) formation (Supplementary Figure S4). *Paenibacillus* S-12 formed mature spores in the DSM medium and microscopic observation showed the DPA accumulation in the centre (Supplementary Figure S5).

### Genome analysis

**Fig. 2:**
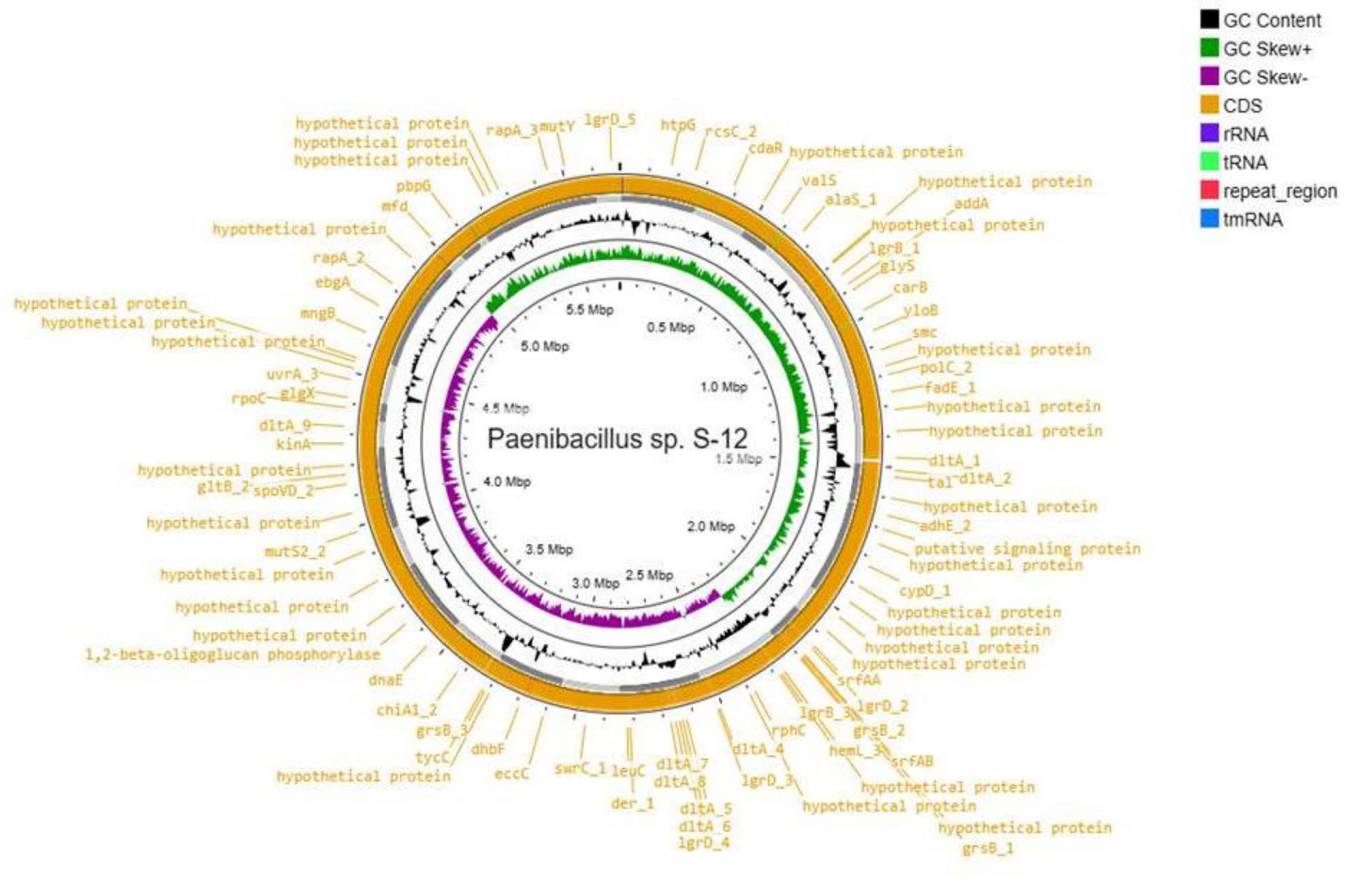
Circular genome map of *Paenibacillus* sp. S-12 constructed by DNA plotter. Rings from inside represent the following: (1) GC content, (2) GC skew (3) CDS features (4) rRNA (5) tRNA, (6) repeat region, (7) positions labels for genome length (Mbp)

**Fig. 3:**
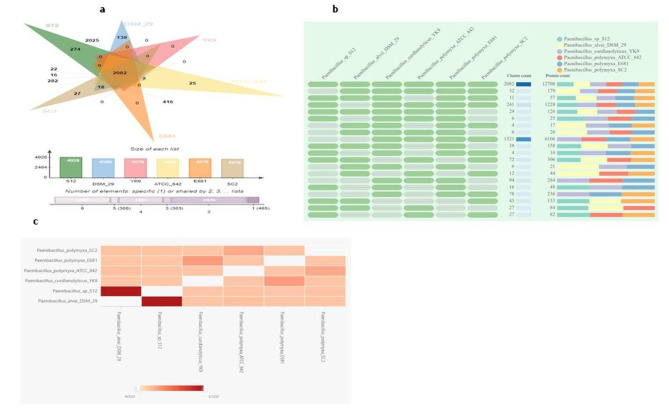
**a.**Comparison of cluster of orthologs groups in five *Paenibacillus* species. The analysis was done by using Orthovenn2 using default parameters with protein sequences of *Paenibacillus* sp. S-12, *P. alvei* DSM29, *P. curdlanolyticus* YK9, *P. polymyxa* ATCC 842, P. *polymyxa* E681 and *P. polymyxa* SC2, **b.** The occurrence table contains groups of gene clusters like cluster count and protein count. Row indicates the orthologous gene cluster for multiple species that summarized as a cell graph and column indicates different closely related bacterial species, C. The pairwise protein sequence comparison for heatmap showing orthologs clusters between S-12 and other closely related strains

The genome sequencing of S-12 using the Illumina sequencing platform generated 1,230,695,800 bp paired-end reads. The *de-novo* assembly of the reads using SPAdes generated twenty seven contigs constituting a circular chromosome of 5.69 Mb (Fig. 2). The genome annotation noted 6068 protein coding genes (CDSs), 27 rRNA, and 15 tRNA in the genome of S-12 (Table 2).Orthovenn2 exhibited that protein clusters shared by all six species were 2082, 308 shared by five species, 1651, 303 and 2826 shared by four, three, and two species, respectively. A total of 465 protein clusters were specific to only a single genome (Fig. 3a). Out of the 465 gene clusters, 274 belonged to *Paenibacillus* sp. S-12, 139 to *P. alvei* DSM29, 25 to *P. polymyxa* ATCC 842, and 27 to *P. polymyxa* SC2. Protein coding gene comparison was performed between *Paenibacillus* sp. S-12 and other five closely related strains. The first pattern shows the gene clusters in the graph, whereas clusters count and total protein count are displayed in second and third pattern of the stacker graph, respectively (Fig. 3b). Similarly, the heat map between S-12 and the other five strains demonstrated overlapping gene clusters in a pair wise pattern (Fig. 3c). The lowest thresholds of gene clusters were observed between *Paenibacillus* sp. S-12 and *P. alvei* DSM 29.

### RAST functional annotation

**Fig. 4:**
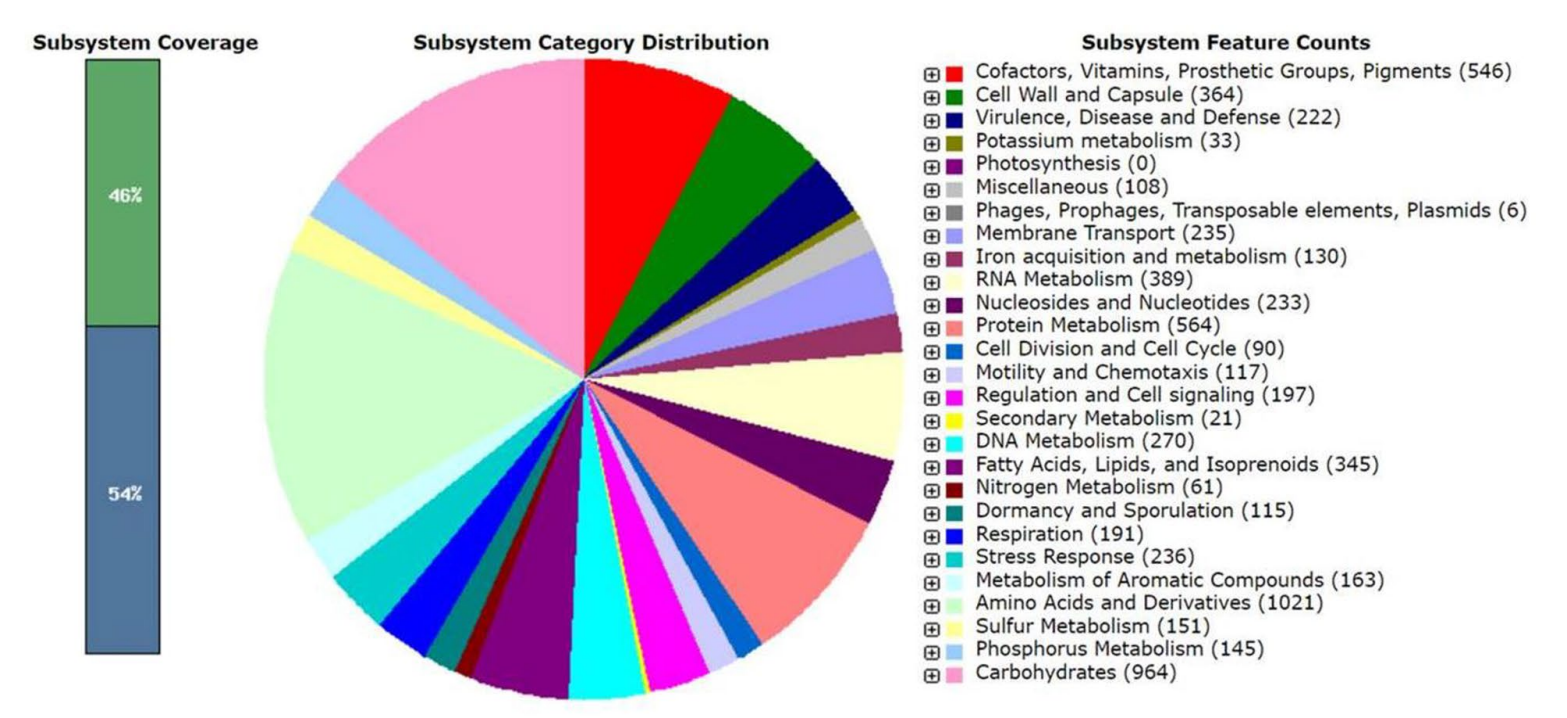
The RAST subsystems distribution in the *Paenibacillus* sp. S-12 genome. The most abundant systems on the category level are shown in the left pie chart, whereas the right column showing the counts of features

Genes were predicted in the S-12 genome using RAST (http://rast.nmpdr.org/) server and subsystem/non-subsystem coverage generated are 46% and 54%, respectively (Fig. 4). The top three subsystem category distributions are amino acids and derivatives (1021 genes), carbohydrate (964 genes) and protein metabolism (564 genes). The other subsystem categories of vitamins & cofactors (546 genes), RNA metabolism (389 genes), cell wall and capsules (364 genes), and fatty acid metabolism (345 genes) were observed (Fig. 4).

RAST-based functional annotation identified the various genes associated with flagellar motor protein, flagellar biosynthesis, and chemotaxis. Among flagellar biosynthesis, we observed the genes FlhA/B and Flil for the flagellar structure formation. Among flagellar motor proteins, genes responsible for flagellar motor rotation protein MotA/B, and genes for flagellar motor switch protein FliM/N were observed. Similarly, chemotaxis-associated genes such as CheA/V/Y were identified (Supplementary Table 4). Among multidrug resistance efflux pumps, RND family MDR membrane protein CmeA/B, outer membrane lipoprotein CmeC, transcription repressor of multidrug efflux pump belonging to acrAB operon, and TetR (AcrR) family were observed. Similarly, the MATE family of MDR efflux pumps belonging to extrusion protein (Na^+^/drug antiporter), toxin extrusion pump YdhE/NorM, and Multidrug-efflux superfamily (MFS) transporter was also observed (Supplementary Table 5).

Genome annotation also identified the various stress-tolerant genes like glutathione redox reaction (15), CoA-disulfide thiol-di-sulfide redox system (1), redox-dependent regulatory proteins (13), rubrerythrin (40), cold shock CspA family protein (8), and DNAK family (33) genes. The other genes include the detoxification stress response (31), flavohaemoglobin (5), sigma B stress response regulation (9), Hfl operons (4), and carbon starvation (8). Various genes related to iron uptake like siderophore enterobactin (2), bacillibactin siderophore (13), iron siderophore, sensor and receptor system (3), and siderophore anthrachelin (7) were identified.

#### Gene ontology

**Fig. 5:**
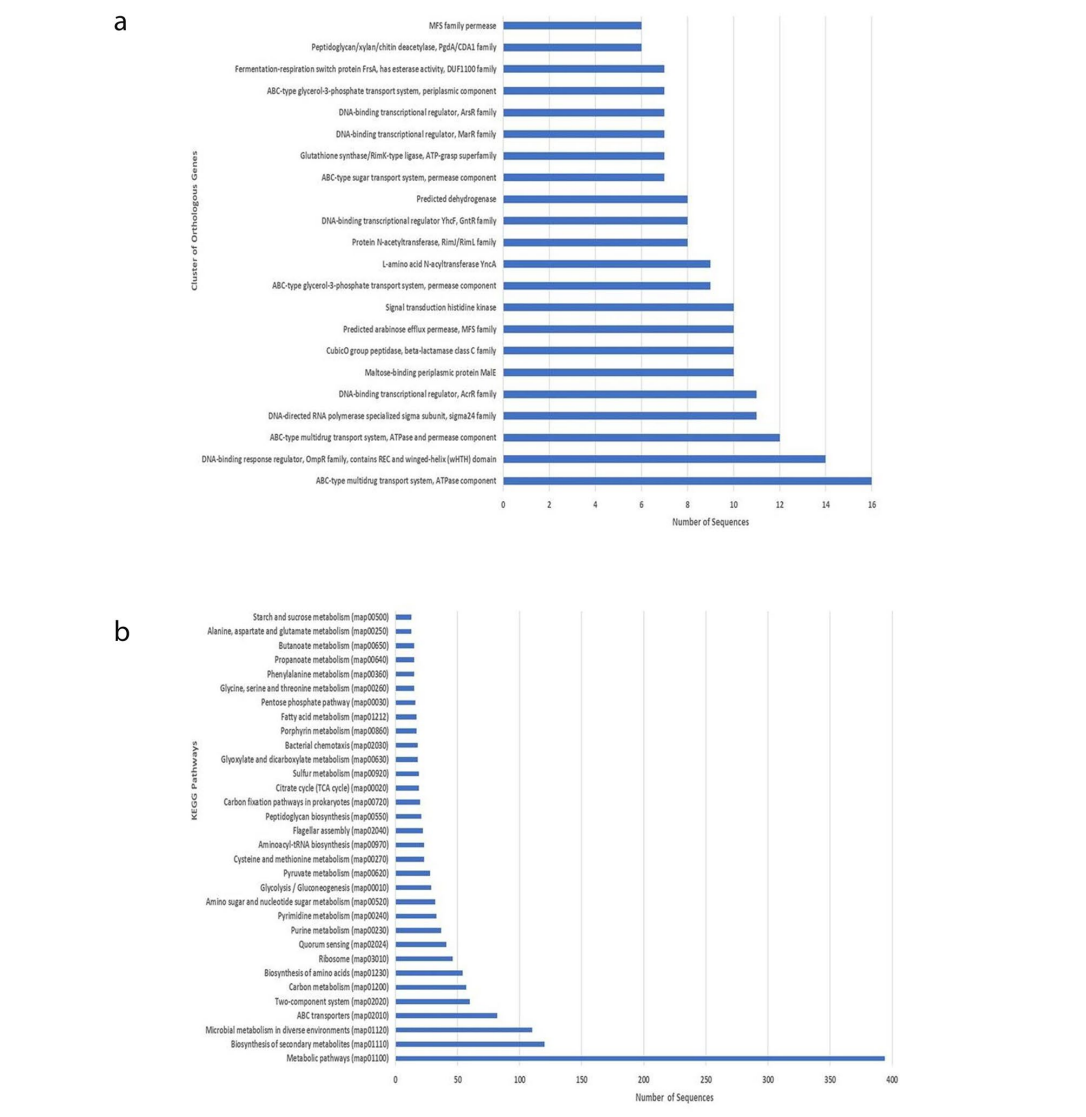
**a** The clusters of orthologus (COGs) analysis in *Paenibacillus* sp. S-12 genome, **b.** The metabolic pathway analysis using KEGG Automatic Annotation Server (KAAS) database. KAAS database is used for functional annotation of genes by BLAST comparisons against KEGG-GENES database

COG analysis predicted the highest number of genes (16) for the ABC-type multidrug transport system, followed by the DNA-binding response regulator (OmpR family, 14) and permease component (12) (Fig. 5a). An equal number of genes (11) belonging to DNA directed RNA polymerase and AcrR family of DNA-binding transcriptional regulators were identified. Similarly, equal numbers (10) of maltose binding protein MalE and beta-lactamase class C family proteins were also observed. It was followed by the equal number of genes (10) for MFS family efflux permease and signal transduction histidine kinase. KEGG analysis showed the various proteins belonging to different metabolic pathways (Fig. 5b). The highest number was recorded for metabolic pathways (390), biosynthesis of secondary metabolites (125) followed by genes (115) responsible for metabolism in diverse environments.

### AMR and VF analysis

**Fig. 6:**
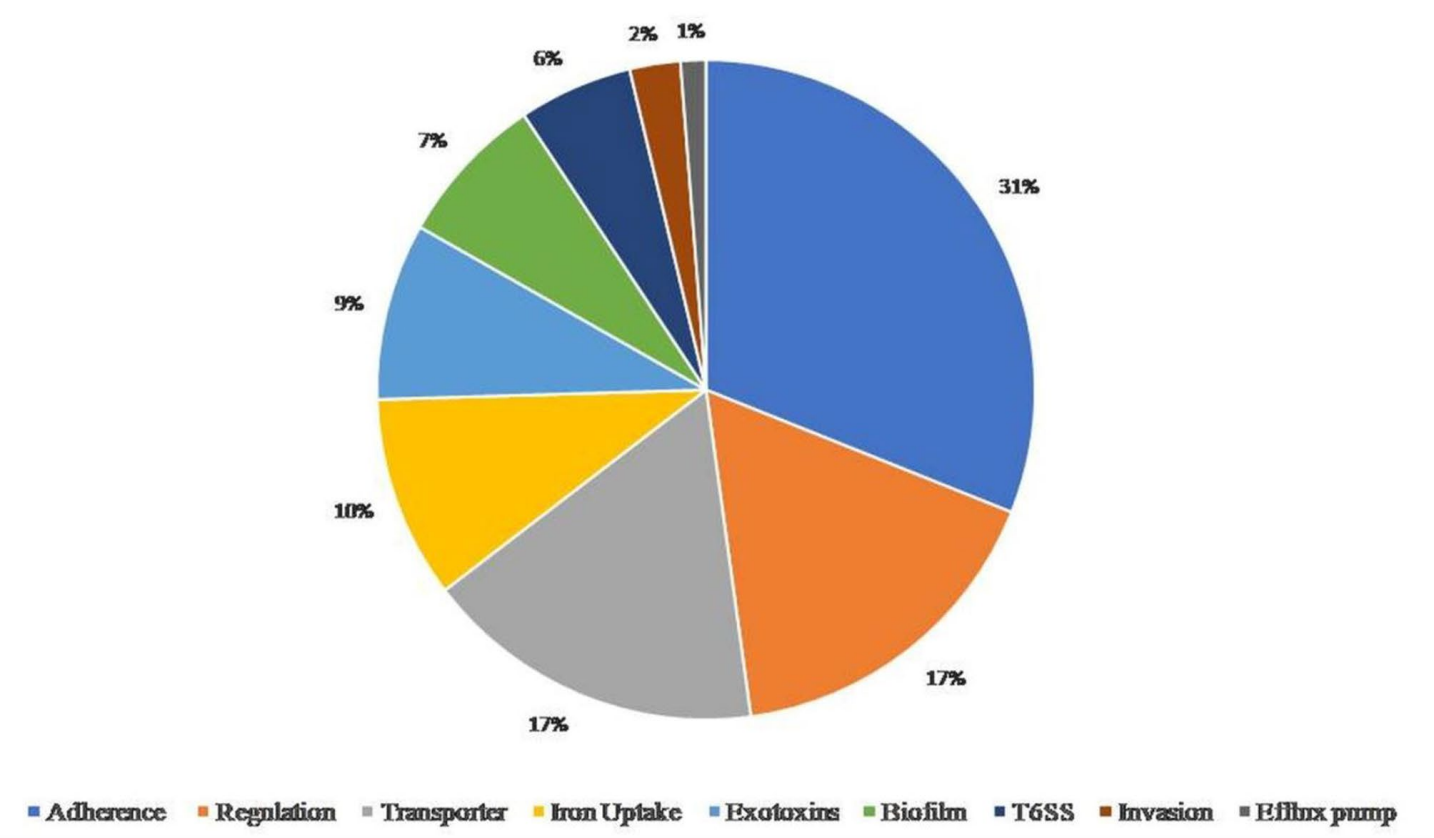
The genome of *Paenibacillus* sp. S-12 was annotated for virulence factor identification using the VFDB database (http://www.mgc.ac.cn/VFs) using the Basic Local Alignment Search Tool (BLASTX) through diamond tool

We observed various AMR genes belonging to the category of major facilitator superfamily (MFS) antibiotic efflux pump, small multidrug resistance (SMR) antibiotic efflux pump, fluoroquinolone-resistant parC, resistance-nodulation-cell division (RND) antibiotic efflux pump and ADC beta-lactamase. Among the different drug class, we observed the genes for macrolide antibiotic, fluoroquinolone antibiotic, cephalosporin, tetracycline antibiotic, fluoroquinolone antibiotic, aminocoumarin antibiotic, lincosamide antibiotic, streptogramin antibiotic, and fosfomycin. The different antibiotic resistance genes with their functional class have been summarized in Supplementary Figure S6 a&b. The VFDB analysis identified various genes related to virulence characteristics such as adherence, invasion, exotoxin, biofilm formation, and transporters etc. (Fig. 6). The highest percentage was noted for adherence (31%), transporters and regulation (17%), iron uptake (10%), exotoxin (9%), and biofilm (7%) etc.

#### Genomic island (GI)

Using Island viewer, GI was identified in the S-12 genome. Predicted GI of *Paenibacillus* sp. S-12 includes various hypothetical proteins, separation proteins, peptidase, and survival proteins (surA) which assist the bacteria in survival in diverse environments (Supplementary Figure S7).

#### Biosynthetic gene clusters

**Fig. 7:**
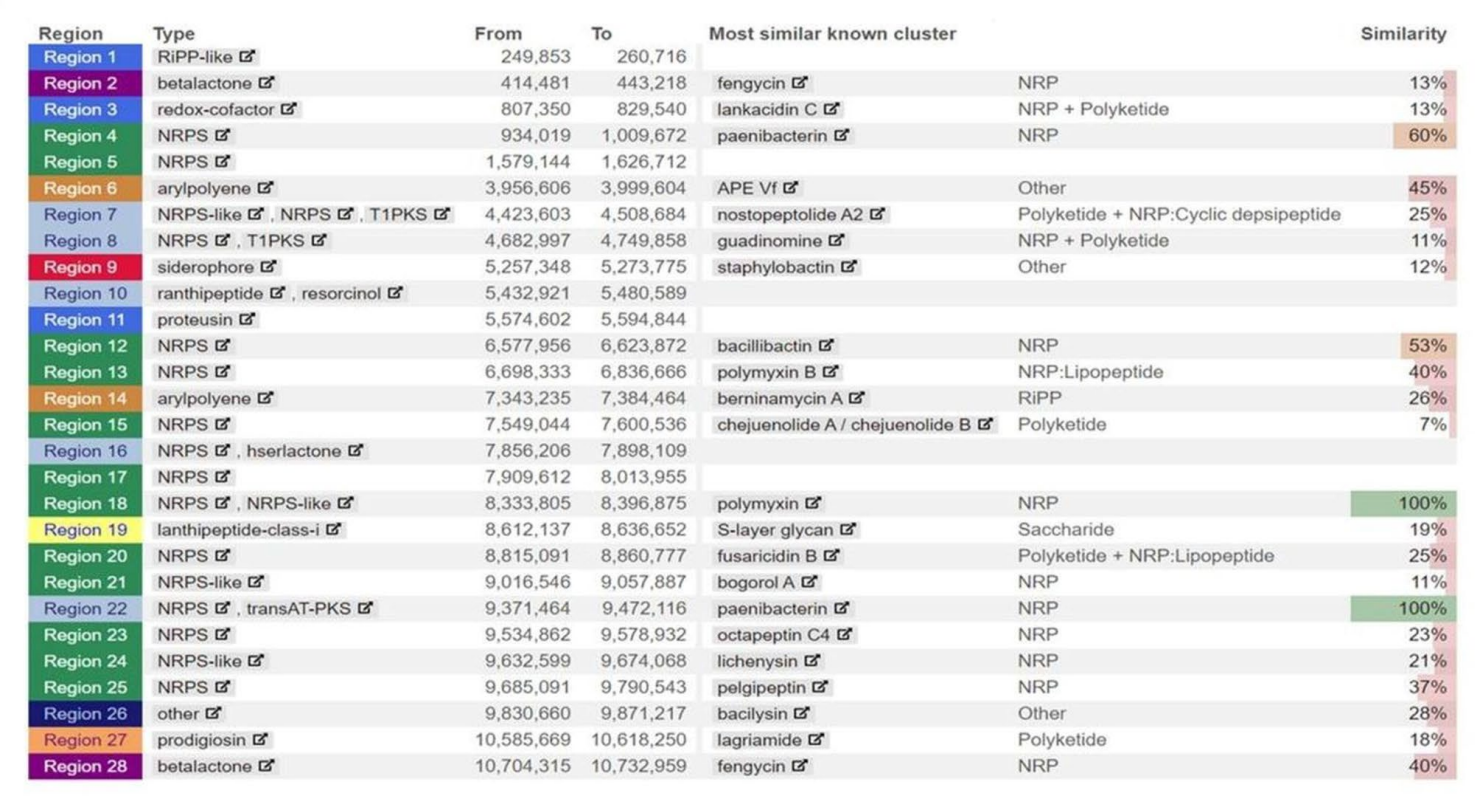
Identification of putative biosynthetic gene clusters (BGCs) using antiSMASH. antiSMASH analysis identified the 28 BGCs in the *Paenibacillus* sp. S-12 genome

The antiSMASH analysis identified genes for antimicrobial peptides (AMPs), secondary metabolite production, NRPSs, and PK synthesis. Various NRPs regions related to paenibacterin, guadinomine, polymyxin B, chejuenolide A/ chejuenolide B, fusaricidin, pelgipeptin, and octapeptin-C4 were identified (Fig. 7). The gene cluster for arylpolyene, siderophore like staphylobactin, lanthipeptide-class of S-layer protein, and betalactone were observed (Fig. 7). Fengycin and paenibacterin are the lipopolysaccharides (LPs) identified through antiSMASH analysis.

#### CAZy carbohydrase analysis

**Fig. 8:**
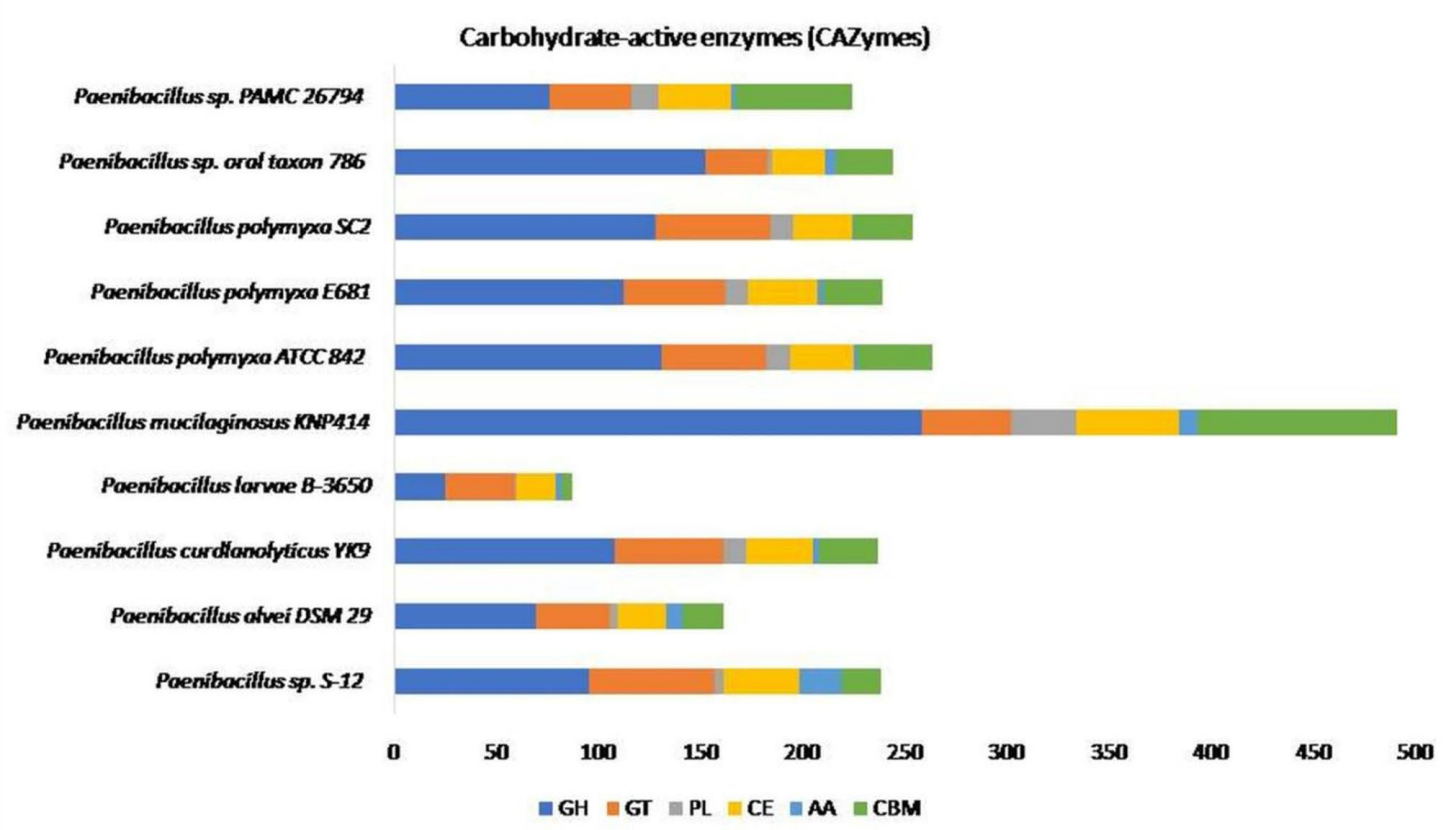
The distribution of CAZyme domain sequences such as Glycoside hydrolases (GHs); Glycosyl transferases (GTs); Polysaccharide lyases (PLs); Carbohydrate esterases (CEs); Auxiliary activities (AAs); and Carbohydrate-binding modules (CBMs) among the *Paenibacillus* sp. S-12 and its closely related species

The CAZy analysis revealed that S-12 has 95 genes for GHs, 62 for GTs, 37 for CEs, and 21 belonging to AAs, whereas 19 and 4 were related to CBMs and PLs, respectively (Table 3). In the GHs group, the higher subcategory was observed for GH29, and GH19, followed by GH20. Among GTS, the major subcategory was observed for GT2 followed by GT4, and GT51. Among CEs, the major subcategory was reported for CE1 followed by CE4, and CE14, whereas in AAs, AA3 showed a higher number followed by AA6, and AA1. Among CBMs, the major subcategory was noted for CBM32 followed by CBM34, and CBM70. Among PLs, the higher subcategory was observed for PL8, and PL4. A total of 2438 CAZyme-encoding sequences were observed among *Paenibacillus* closely related species tested in the present study. The number of CAZyme-encoding sequences was the highest (n = 491) and the lowest (n = 87) for *P. mucilaginosus* KNP414 and *P. larvae* B-3650, respectively (Fig. 8).

**Table 1:** Probiotic features observed following genome analysis of S-12

*Bile resistance*	
Ornithine decarboxylase	Enhancement of intracellular Ph
Sodium bile acid symporter	Bile acid uptake
*Acid tolerance*	
Alanine dehydrogenase	Pumps proton out of cell
Arginine decarboxylase	Proton consumption
Agmatine deiminase	Transform agmatine to putresine
Arginine deiminase	Transform arginine to citrulline
Arginine/Ornithine antiporter	Imports arginine and exports ornithine
Formate dehydrogenase	Pumps proton out of cell
F0F1 - ATP synthase	Proton translocation
Glutaminase	Converts glutamine to glutamate
Glycine betaine transport system	Osmoregulation
Glutamate decarboxylase	Transform glutamate to GABA
Lactate dehydrogenase	Maintenance NAD^+^/NADH balance
Ornithine transcarbamylase	Convert citrulline to Ornithine and carbamyl phosphate
Sodium hydrogen antiporter	Proton transporter

**Table 2:** Genome feature of *Paenibacillu*s sp. S-12

Annotated genome features	
Contigs	27
GC-content	46.5%
Genome size	5.69 Mb
CDS	6,098
tRNA	15
rRNA	27
Hypothetical proteins	2150
Proteins with GO assignment	3,477
Proteins with pathway assignment	2209

**Table 3:** The distribution of CAZymes in *Paenibacillu*s sp. S-12

Function Class	Number	Family Number
GH	95	GH1(2), GH2(1), GH3(7), GH4(2), GH8(1), GH13 (4), GH15(1), GH18(12), GH19(3), GH20(3), GH23(6), GH24(1), GH25(2), GH29(4), GH31(1), GH35(1), GH38(1), GH39(2), GH46(1), GH57(1), GH63(1), GH85(3), GH87(1), GH88(2), GH92(1), GH94(1), GH95(1), GH103(2), GH108(2), GH109(13), GH112(1), GH125(2), GH126(1), GH130(1), GH136(1), GH151(1), GH153(1), GH154(1), GH170 (2)
CBM	19	CBM2(1), CBM5(1), CBM(1), CBM9(2), CBM32(7), CBM34(2), CBM35(1), CBM41(1), CBM54(1), CBM70(2)
GT	62	GT1(2), GT2(23), GT3(1), GT4(19), GT17(1), GT19(1), GT20(1), GT25(1), GT26(1), GT28(5), GT30(1), GT51(6)
AA	21	AA1(3), AA3(7), AA4(1), AA6(5), AA7(2), AA10(2), AA12(1)
CE	37	CE1(11), CE3(1), CE4(15), CE7(3), CE9(1), CE11(1), CE14(5)
PL	4	PL6(1), PL8(2), PL31(1)

### Pan genome-core genome analysis

**Fig. 9:**
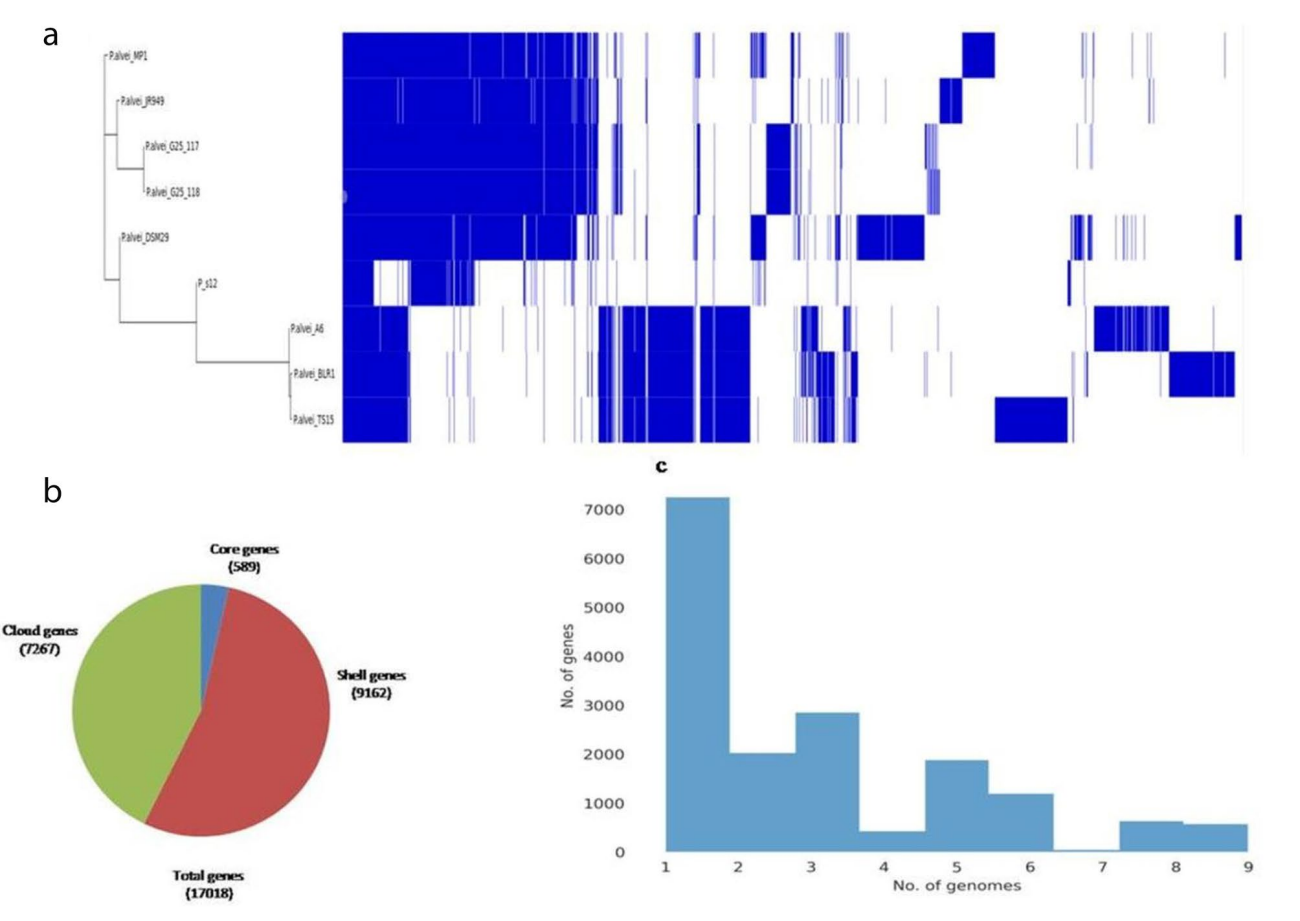
**a.**The matrix illustrating the presence/absence of genes in selected genome, the clustering of tree is shown on the left side, **b.** The pie chart shows the proportion of core, shell, and cloud genes **c.** The gene frequency plot demonstrating the distribution of genes per genome

The pangenome analysis was done using nine closely related genomes of non-type strains in the Roary tool. The generated matrix showed the presence/absence profile of genes in selected strains. The most closely related strains of *Paenibacillus* S-12 belonged to *P. alvei* A6, P. *alvei* BLR1 and *P. alvei* TS-15 (Fig. 9a). Gene presence/absence profiles for *P. alvei* A6, P. *alvei* BLR1 and *P. alvei* TS-15 were similar, while *Paenibacillus* S-12 displayed a distinct profile from other strains belonging to the same cluster. The pangenome of all nine selected strains contains 17,018 genes, of which the core genome represents 589 genes (18%), the shell genome represent 9162 genes (43%), and the cloud genome represent 7267 genes (38%) (Fig. 9b). On average each strain contained 105 unique genes which correspond to approximately 3% of each genome (Fig. 9c).

## Discussion

The S-12 strain was identified as *Paenibacillus* sp. based on the sequence analysis of the 16 S rRNA gene. The *Paenibacillus* spp. exhibits environmental survival and increases its population in various ecological niches. However, the evidence at the genomic level is still lacking. A previous report illustrated that bacterial strains with larger genome exhibit more adaptability to complex habitats as larger genomes bear more genes for metabolism and stress tolerance [63]. However, the other studies demonstrated that even a small bacterial genome might also show more competitive, advantages in energy saving, and reproductive efficiency [64, 65]. In the present study, we explore the in-depth genomic analysis to unravel the information about probiotic features, multidrug efflux pumps, transporter genes, presence of antimicrobial, virulence genes, and stress-protectant etc.

In the gut, bile salts exert several deleterious effects like the disruption of the bacterial membrane, denaturation of the proteins, chelation of various ions like iron and calcium, and also modify the eukaryotic gene expression related to the host immunity and defence [66]. To resist these deleterious effects, microorganism evolves defence mechanisms like bile efflux, hydrolysis of bile salts, induction of stress proteins, and reorganization of metabolic pathways [67, 68]. In the test isolate, the various genes encoding bile and sodium symporter, ABC transporter, arginine, and ornithine decarboxylase were noted which might help the bacterium to survive in presence of salts [69, 70]. Bile salts or bile salt hydrolase breaks the complex bile acids and other molecules such as glycine/taurine to allow its diffusion into the cell, leading to increased intracellular acidification [70]. Similarly, arginine and ornithine decarboxylase catalyzes the decarboxylation of arginine/ornithine to putrescine, thereby increasing the intracellular pH [71, 72]. Under these circumstances, F0F1- ATP synthase translocates the protons out and therefore, favours the acid and bile tolerance [73, 74]. It was observed that thepresence of F0F1- ATP synthase and bile-salt symporter minimized the toxic effects of acid and bile effects in probiotic bacteria [69, 70].

The test isolate showed biofilm formation, which can help the bacterium for colonization, and also protection of its host plant against stressors. A previous study showed that biofilm forming plant growth promoting rhizobacterium *P. polymyxa* colonized the plant roots with the formation of biofilm, and further improved plant resistance to biotic and abiotic stressors [75]. Another study showed that exoglycans producing bacterium *P. polymyxa* strain 1465 favour the colonization of the bacterium to wheat roots [76].

Most of the *Paenibacillus* spp. are non-pathogenic, however *P. alvei*, *P. thiaminolyticus* and *P. sputa* showed pathogenicity in terms of respiratory andurinary tract infection, and bacteremia in hemodialysis patients [77–79]. The test isolate showed resistance to several antibiotics. The resistance pattern highlights the possible presence of resistant determinants in the S-12. Therefore, we explore the various genomic features in the test isolate through genome sequencing and annotation. The *Paenibacillus* sp. S-12 genome features many multidrug efflux transporters conferring resistance to many antibiotics. These proteins particularly enhance the efflux, diffusion, and other bacterial defense mechanism against xenobiotics [80, 81]. We noted chloramphenicol acetyltransferase, vancomycin resistance protein vanX, vanH, vanA, & vanW, and vancomycin resistance regulator vanR protein [82]. Additionally, a bacteriocin resistance gene, tetracycline resistance protein TetM, TetO, TetP, TetW, and oxytetracycline resistance protein OtrA were identified. The genome contains some other genes conferring resistance to fluoroquinolones, β-lactams, and dihydrofolate reductase-A inducing resistance to trimethoprim as well as resistance genes to streptothricin [83]. Moreover, the isolate contains the multidrug resistance efflux proteins belonging to the family of RND efflux system, MATE family of antimicrobial extrusion protein, MFS family of multidrug-efflux transporter, and Mex family of multidrug efflux transporteretc. Additionally, the isolate contain the genes inducing resistance to acriflavine, outer membrane multidrug efflux pump, drug transport regulator NfxB, and PmrA multidrug resistance efflux pumps. The present work is the first report showing the detailed characterization of the multidrug efflux system in the *Paenibacillus* bacterial strain.

The genome of *Paenibacillus* sp. S-12 features several ABC transporters like oppA (oligopeptide ABC transporter), oppB/C (oligopeptide transport system permease protein), and oppC/D (oligopeptide transport ATP binding protein). In bacteria, the signal recognition particles (SRP) initiate the co-translational protein targeting to the plasma membrane by binding to the N-terminal signal sequence from the translating ribosome. In the strain S-12, we observed the three genes related to SRP such asFtsY (signal recognition particle receptors protein), Ffh (signal recognition particle subunit ffh), SRP-AP (signal recognition particle associated protein), and twin-arginine translocation system TatA/B/C/E (twin-arginine translocation protein systems).

We also identified various metal transporters following annotation of the S-12 genome. In Mg (magnesium)-transporters, various genes like mgtA (Mg^++^ transport ATPase protein-P), mgtC (Mg^++^ transport ATPase protein-C), mgtE (Mg-Co-Ni transporters), corA( Mg/Co transport protein), corC (Mg/Co efflux protein), and cat (cation transporting ATPase) were identified. In the copper transporter systems, the genes identified as YcnL (reductase &disulfide isomerise in Cu uptake), YcnK (transcriptional repressors of Cu-uptake), CopA (Cu-translocating P-type ATPase), CopC/D (coper resistance protein), YcnI (membrane protein in Cu uptake), CsoR (repressors of Cu-operon), and CopZ (copper chaperones) [84]. The various genes responsible for nickel (Ni) transport such as NikA (Nickel ABC transporter), NikB/C (Nickel permease protein), NikE (Nickel transport ATP-binding protein), and NikR (Nickel responsive regulator) were identified. Similarly, for cobalt transporters, genes like CbtA/C (cobalt transporter), CbtF (cobalt ABC transporter periplasmic component), and CbtJ/K/L (cobalt ABC transporter) were noted [84]. Besides, genes related to arsenic efflux pumps (arsA, arsB, ACR3) as well as arsenate reductase (arsC, ArrA, ArrB, ArrS) were identified. Among the other metals, we noted the genes conferring resistance to cadmium transport (cadA), cadmium efflux system (cadC), and cadmium resistance protein (cadD). The genes conferring resistance to chromium compounds like chromate resistance protein (ChrI, ChrB), chromate transport protein (ChrA), rhodanese-like protein (ChrE), and superoxide dismutase like protein (ChrF) was identified [84]. Among the *Paenibacillus* strains, *Paenibacillus* sp. LYX-1 showed cadmium resistance, and exhibited biocontrol activity [84].

We identified the Type I secretion system that secretes RTX-like adhesion required for auto-aggregation and biofilm formation. The gene like LapB (Type I secretion system ATPase), LapC (membrane fusion protein), LapE (outer membrane component), LapD (membrane bound c-di-GMP receptor), LapP (transglutaminase like cystine protenease), LapL (peptidoglycan associated lipoproteins), and RTX (T1SS-secreted agglutinins) were identified [85]. The presence of flagella, flagellar-associated protein and flagellar regulatory protein helps the bacteria for their colonization and stress survival. Through genome analysis, we identified the flagellin protein (FlaAB), flagellar biosynthesis protein (FlhA/B), flagellar motor protein (MotA/B), and flagellar motor switch protein (FliM/N). Moreover, proteins related to chemotaxis such as CheA/V/Y were also notified. Previous study demonstrated the presence of flagellum in *Paenibacillus* sp. NAIST15-1, which showed the increased transcription of flagellar genes and hyper-flagellation when transferred from liquid to solid medium [85].

Our genome analysis showed the presence of an NRPS cluster with known predicted functions and a RIPP cluster with unknown products. The discovery of NRPS-lipopeptide highlighted to be attractive pharmaceutical and/ or industrial products. Gene clusters responsible for AMPs, polyketide, polymyxin, and fusaricidin etc., were identified with potent antimicrobial activity [86]. Members of the polyketide group exhibit strong antagonistic activity against food borne pathogens [87]. Presence of these diverse AMPs may increase the ability of *Paenibacillus* sp. S-12 to fight against pathogenic microorganisms.

The microorganism responsible for plant cell wall degradation plays an essential role inthe recycling of photosynthetically fixed carbon, however, only few microbes are capable to hydrolyze the complex cellulose. Among *Paenibacillus* genus, *P. polymyxa* A18 showed higher cellulolytic and hemicellulolytic activities [44]. In this study, we explored the genome of strain S-12 to identify the genes responsible for complex carbohydrate degradation. The carbohydrate enzymes CAZymes are involved in the synthesis as well as the breakdown of complex carbohydrate polymers. The identified CAZymes like GHs and GTs perform the hydrolysis of glycosidic bonds and are commonly noted in *Paenibacillus* species [88]. The other CAZymes like AAs, PLs, and CBMs are involved in the degradation of several compounds including biopolymers [89]. The improved bioinformatics approach has allowed the identification of gene distribution among its closest relatives by comparative genomics. Gene comparison as well as pengenome exploration leads to discovery of genes involved in strain diversification [90]. The improved genome sequencing approaches pave the way for pangenome investigations in bacteria [91]. The pangenome analysis indicates the S-12 strain harbor many unique genes which are not shared by other strains and thereby gene pool size would increase further increased number of genomes incorporated in the analysis. The open pan-genome indicates that *Paenibacillus* have the tendency to change its genomic content to adapt to the environment.

## Conclusion

Overall, the current findings provide the information about the *Paenibacillus* sp. S-12 genome that might have acquired or possessed genome features to survive under diverse environmental conditions. The S-12 strain showed probiotic traits essential to thrive through the gastrointestinal transit and also possessed respective genes, making it a strong candidate for probiotics and industrial applications. The presence of antimicrobial genes harnessed by the strain illustrates its ability to mitigate the intestinal pathogens. Moreover, the presence of various plant growth-promoting genes or gene clusters shows its potential to enhance the plant growth and further development of microbial biopesticides.

## Electronic supplementary material

Below is the link to the electronic supplementary material.

Supplementary Material 1

## Data Availability

The datasets generated and/or analysed during the current study are available in the Genbank (https://www.ncbi.nlm.nih.gov/genbank/) under Bioproject and Biosample accession no. PRJNA861075 and SAMN29881974, respectively. The raw illumina data were submitted to the NCBI Sequence Read Archive (SRA) under accession number SRR20556255. The genome sequence was submitted to NCBI and accession no. JASIUF000000000 was assigned.

## Abbreviations

*ORF*: Open reading frame
*CAZymes*: Carbohydrate-active enzyme
*BGCs*: Biosynthetic gene clusters
*AFB*: American Foulbrood, *HGT*:Horizontal gene transfer
*AMPs*: Antimicrobials proteins
*NRPSs*: Nonribosomal peptide synthetases
*PKSs*: Polyketide synthase
*RiPPs*: Ribosomally synthesized and post-translationally modified peptide
*PBS*: Phosphate buffer saline
*IMViC*: Indole, Methyl Red, Voges Proskauer
*ZOI*: Zone of inhibition
*SEM*: Scanning electron microscopy
*TSB*: Tryptic soya broth
*CV*: Crystal violet
*DSM*: Difco sporulation medium
*tRNA*: transfer RNA
*rRNA*: ribosomal RNA
*COG*: Clusters of orthologous groups
*HMM*: Hidden Markov Model
*RAST*: Rapid annotations using subsytems technology
*MFS*: Major facilitator superfamily
*RND*: Resistance-nodulation-cell division
