# Supplementary material for: Characterization and in-depth genome analysis of a halotolerant probiotic bacterium Paenibacillus sp. S-12, a multifarious bacterium isolated from Rauvolfia serpentina
Source: BMC Microbiol. 2023 Jul 18;23:192. doi: 10.1186/s12866-023-02939-1 (PMC10353221; doi:10.1186/s12866-023-02939-1)
Supplement: Supplementary file 1 — Supplementary Material 1 [file 12866_2023_2939_MOESM1_ESM.docx]

**Supplementary information**

**In-depth genome analysis of halotolerant *Paenibacillus* sp. S-12, a multifarious bacterium isolated from *Rauvolfia serpentina***

Rajnish Prakash Singh^1^*, Kiran Kumari^1^, Parva Kumar Sharma^2^, Ying Ma^3^

**Corresponding Author**: Rajnish Prakash Singh ([manasrajnish2008@gmail.com](mailto:manasrajnish2008@gmail.com))

**Affiliations:** ^1^Department of Bioengineering and Biotechnology

Birla Institute of Technology, Mesra, Ranchi, Jharkhand, India

Pin- 835215

^2^Department of Plant Sciences and Landscape Architecture, University of Maryland, College Park, MD-20742

^3^ College of Resources and Environment, Southwest University, Chongqing, China

**Supplementary Table 1 Biochemical characteristic feature of *Paenibacillu*s sp. S-12**

**Characteristic (s) Activity Carbohydrate Activity**

Gram reaction + Sodium gluconate -

Catalase + Glycerol +

Indole - Salicin -

MR - Dulcitol -

VP + Inositol +

Amylase - Sorbitol +

Lipase + Mannitol -

Pectinase - Adonitol -

Catalase + Inulin +

Cellulase + Arabitol -

Temp. tolerance (°C) 30-40 Erythritol -

Salt tolerance (%) 6% Citrate +

pH 5-9 α-Methyl-D-glucoside -

Swimming + L-Arabinose -

Swarming + Rhamnose +

Twiching + Cellobiose +

**Carbohydrate Activity** Melezitose +

Lactose + α-Methyl-D-mannoside -

Xylose + Xylitol -

Maltose + ONPG +

Fructose + Esculin hydrolysis +

Dextrose - Mannose +

Sucrose + D-Arabinose +

Galactose + Malonate utilization +

Raffinose _-_ Trehalose +

**Supplementary Table 2 Antibiotic sensitivity test of *Paenibacillus* sp. S-12**

| **Antibiotics** |  |
| --- | --- |
|  | **S-12 (ZOI)** |
| Streptomycine (S10) | 22 mm |
| Ampicillin (Amp10) | 12 mm |
| Gentamicin (GEN10) | 21 mm |
| Tetracycline (TE30) | 16 mm |
| Kanamycin (K30) | 0 mm |
| Erythromycin (E15) | 23 |
| Ciprofloxacine (CIP5) | 22 mm |
| Fluconazole (FLC25) | 15 mm |
| Vancomycin (VA30) | 25 mm |
| Voriconazole (VRC1) | 0 mm |

**Supplementary Table 3 Antagonistic activity of S-33**

**Bacteria Activity Zone of inhibition (mm)**

*Escherichia coli* +++ 16.40±0.30

*Staphylococcus aureus* ++ 13.70±0.22

*Bacillus subtilis* ++ 12.70±0.19

*P. aeruginosa* +++ 17.09±0.35

**Fungal species**

*Aspergillus niger* +++ 16.50±0.21

*Microsporum gypseum* +++ 17.10±0.29

*H. gypsium* ++ 13.50±0.31

*Penicillium citrium* ++ 13.10±0.22

+++ good; ++ moderate, + poor; (±)denote standard deviation; NA no activity

**Supplementary Table 4 Observation of flagellar biosynthesis, flagellin protein and other chemotaxis –associated genes in genome of S-12**

| Gene | Functional role |
| --- | --- |
| flgA | Flagellar basal-body P-ring formation protein |
| flgB | Flagellar basal-body rod protein |
| flgC | Flagellar basal-body rod protein |
| flgD | Flagellar basal-body rod modification protein |
| flgE | Flagellar hook protein |
| flgF | Flagellar basal-body rod protein |
| flgG | Flagellar basal-body rod protein |
| flgH | Flagellar L-ring protein |
| flgI | Flagellar P-ring protein |
| flgJ | Flagellar protein |
| flgK | Flagellar hook-associated protein |
| flgL | Flagellar hook-associated protein |
| flgN | Flagellar biosynthesis protein |
| flhA | Flagellar biosynthesis protein |
| fliE | Flagellar hook-basal body complex protein |
| fliF | Flagellar M-ring protein |
| fliG | Flagellar motor switch protein |
| fliH | Flagellar assembly protein |
| fliI | Flagellum-specific ATP synthase |
| fliJ | Flagellar protein |
| fliK | Flagellar hook-length control protein |
| fliM | Flagellar motor switch protein |
| fliN | Flagellar motor switch protein |
| fliP | Flagellar biosynthesis protein |
| fliQ | Flagellar biosynthesis protein |
| fliR | Flagellar biosynthesis protein |
| fliZ | Flagellar biosynthesis protein |
| fliC | Flagellar biosynthesis protein |
| fliD | Flagellar hook-associated protein |
| fliS | Flagellar biosynthesis protein |
| fliT | Flagellar biosynthesis protein |
| fliO | Flagellar biosynthesis protein |
| flhB | Flagellar biosynthesis protein |
| flhF | Flagellar biosynthesis protein |
| RP-Sigma | RNA polymerase sigma factor for flagellar operon |
| fleN | Flagellar synthesis regulator |
| motA | Flagellar motor rotation protein |
| motB | Flagellar motor rotation protein |
| flaA | Flagellin protein |
| flaB | Flagellin protein |
| rpoD | RNA polymerase sigma factor |
| flhE | Flagellar protein |
| flhC1 | Flagellar biosynthesis protein |
| flhD2 | Flagellar biosynthesis protein |
| fliA | Flagellar biosynthesis protein |
| flgM | Flagellar biosynthesis protein |
| flhD | Flagellar transcriptional activator |
| flhC | Flagellar transcriptional activator |
| fliX | Flagellar trans-acting factor |
| flbD | Flagellar protein |
| flbB | Flagellar protein |
| NegReg2 | Negative regulator of flagellin synthesis |
| NegReg | Negative regulator of flagellin synthesis |
| flaG | Flagellin protein |
| flaC | Flagellin protein |
| flaD | Flagellin protein |
| flaE | Flagellin protein |
| flaF | Flagellin protein |
| fleQ | Flagellar regulatory protein |
| rpoN | RNA polymerase sigma-54 factor |
| hook3 | Flagellar hook-associated protein 3 |
| fleS | Flagellar sensor histidine kinase |
| fliL | Flagellar biosynthesis protein |
| motX | Sodium-type flagellar protein |
| motY | Sodium-type flagellar protein |
| moyY-pre | Sodium-type flagellar protein motY precursor |

**Supplementary Table 5 observation of multidrug resistance efflux pumps, RND, MFS and MATE family of MDR efflux system**

| CmeA | RND efflux system, membrane fusion protein CmeA |
| --- | --- |
| CmeB | RND efflux system, inner membrane transporter CmeB |
| CmeC | RND efflux system, outer membrane lipoprotein CmeC |
| AcrR | Transcription repressor of multidrug efflux pump acrAB operon, TetR (AcrR) family |
| TetR | Transcription regulator of multidrug efflux pump operon, TetR (AcrR) family |
| Reg | Probable transcription regulator protein of MDR efflux pump cluster |
| MATE_all | Multi antimicrobial extrusion protein (Na(+)/drug antiporter), MATE family of MDR efflux pumps |
| YdhE/NorM | Multidrug and toxin extrusion (MATE) family efflux pump YdhE/NorM |
| YdhE/NorM? | Multidrug and toxin extrusion (MATE) family efflux pump YdhE/NorM, homolog |
| MFS | Multidrug-efflux transporter, major facilitator superfamily (MFS) |
| VNT | Vesicular neurotransmitter transporter |
| MacA | Macrolide-specific efflux protein MacA |
| MacB | Macrolide export ATP-binding/permease protein MacB |
| OML | RND efflux system, outer membrane lipoprotein, NodT family |
| TolC_14 | Type I secretion outer membrane protein, TolC precursor |
| MtrF | Multidrug efflux pump component MtrF |
| RND | Membrane fusion protein of RND family multidrug efflux pump |
| AcrB | Acriflavin resistance protein |
| OM | Probable outer membrane component of multidrug efflux pump |
| MexD | Multidrug efflux RND transporter MexD |
| MexC | Multidrug efflux RND membrane fusion protein MexC |
| NfxB | Transcriptional regulator NfxB |
| PmrA | Multidrug resistance efflux pump PmrA |

**Supplementary Figure S1** Morphological analysis of *Paenibacillus* sp. S-12 by the scanning electron microscopy (SEM) analysis.

**Supplementary Figure S2** Test of motility (swimming, swarming, twitching) shown by the *Paenibacillus* sp. S-12.

**Supplementary Figure S3** Test of survival shown by *Paenibacillus* sp. S-12 under different pH (2 to 7) and different bile salt (0.2 to 0.4%) concentration.

**Supplementary Figure S4** Test of biofilm formation by the *Paenibacillus* sp. S-12 at different temperature.

**Supplementary Figure S5** Endospore formation was checked in Difco sporulating medium (DSM) and observed under phase-contrast microscope.

**Supplementary Figure S6a** The CARD analysis of *Paenibacillus* sp. S-12 genome for the mining of various drug efflux family system, **b.** Determination of various class of antibiotic resistance.

**Supplementary Figure S7** Genomic island prediction in *Paenibacillus* sp. S-12 genome by Island Viewer2 tool.
